# Diagnosing Primary Sclerosing Cholangitis in Children

**DOI:** 10.7759/cureus.8334

**Published:** 2020-05-28

**Authors:** Iqtadar Seerat, Muhammad Atique, Eitzaz ud din Khan, Ambreen Akram, Amjad Iqbal

**Affiliations:** 1 Pediatric Gastroenterology & Hepatology, Pakistan Kidney and Liver Institute and Research Center, Lahore, PAK; 2 Histopathology, Pakistan Kidney and Liver Institute and Research Center, Lahore, PAK; 3 Anesthesiology, Pakistan Kidney and Liver Institute and Research Center, Lahore, PAK; 4 Pediatric Gastroenterology, Pakistan Kidney and Liver Institute and Research Center, Lahore, PAK; 5 Radiology, Pakistan Kidney and Liver Institute and Research Center, Lahore, PAK

**Keywords:** primary sclerosing cholangitis, chronic liver disease, children

## Abstract

Primary sclerosing cholangitis (PSC) is a rare chronic cholestatic liver disease in children caused by chronic inflammatory process affecting either intrahepatic or extrahepatic bile ducts. Although it is infrequent, incidence is increasing worldwide, may be due to more awareness, understanding and expertise in managing children with chronic liver disease (CLD). In the developing world like Pakistan where resources and expertise are limited, very few tertiary centers are equipped to manage CLD in children.

This case report is about a teenage child who presented to us with decompensated CLD in the form of jaundice and ascites. In our center, after a much needed workup he was commenced on appropriate treatment for PSC. After six months of treatment, he has managed to clear jaundice. The liver synthetic functions have improved with normal coagulation profile. His MELD (model for end-stage liver disease) score, which has come down from 19 to 9, delays liver transplant (LT) for years, which remains the best available treatment. MELD is a scoring system to assess the severity of CLD and remains an important tool to determine the outcome and ranking for receipt of an LT. Subsequently, he developed colitis and colonoscopy confirmed lymphocytic colitis (LC), which is a rare association of PSC.

## Introduction

Primary sclerosing cholangitis (PSC) is manifested by slow and progressive inflammation of intrahepatic or extrahepatic bile ducts and usually terminates in cirrhosis, portal hypertension and liver failure. It is rare in children, with an incidence and prevalence of 0.2 and 1.5 cases per 100,000 children, respectively [1]. It can occur at any age, but is more common in adults.

The diagnosis depends on clinical presentation, laboratory investigations, ultrasound (U/S) of liver, magnetic resonance cholangiopancreatography (MRCP) and liver biopsy with typical cholangiographic findings. 

We present a case report on a 13-year-old boy whom we diagnosed with PSC with the help of appropriate investigations. Unfortunately, he was misdiagnosed as a case of Wilson's disease and hence received improper treatment for months in his local district general hospital, which resulted in worsening of disease. Here we describe our systematic approach to investigate PSC and associated gastrointestinal complications to avoid any delay in initiating treatment.

## Case presentation

A 13-year-old boy presented to us with jaundice and abdominal distension for one year. No history of itching and weight loss. There is no past medical history of any significant diseases. His parents are first cousins and no one else is affected with chronic liver disease (CLD) in immediate family. His growth has been satisfactory.

His clinical examination exhibited positive jaundice, and the abdominal examination confirmed mild splenomegaly and ascites. The rest of his systemic examination was normal.

We completed the necessary workup for CLD. The liver function tests (LFTs) were deranged. The complete blood count showed thrombocytopenia and the international normalized ratio (INR) was prolonged.

The renal functions and serum electrolytes were normal. The serum study for autoimmune screen, hepatitis B and C screen, and immunoglobulins level was unremarkable. The serum ceruloplasmin, copper and 24-hour urinary copper excretion after oral penicillamine challenge were normal. There was no evidence of Kayser-Fleischer rings in his eyes.

**Figure 1:**
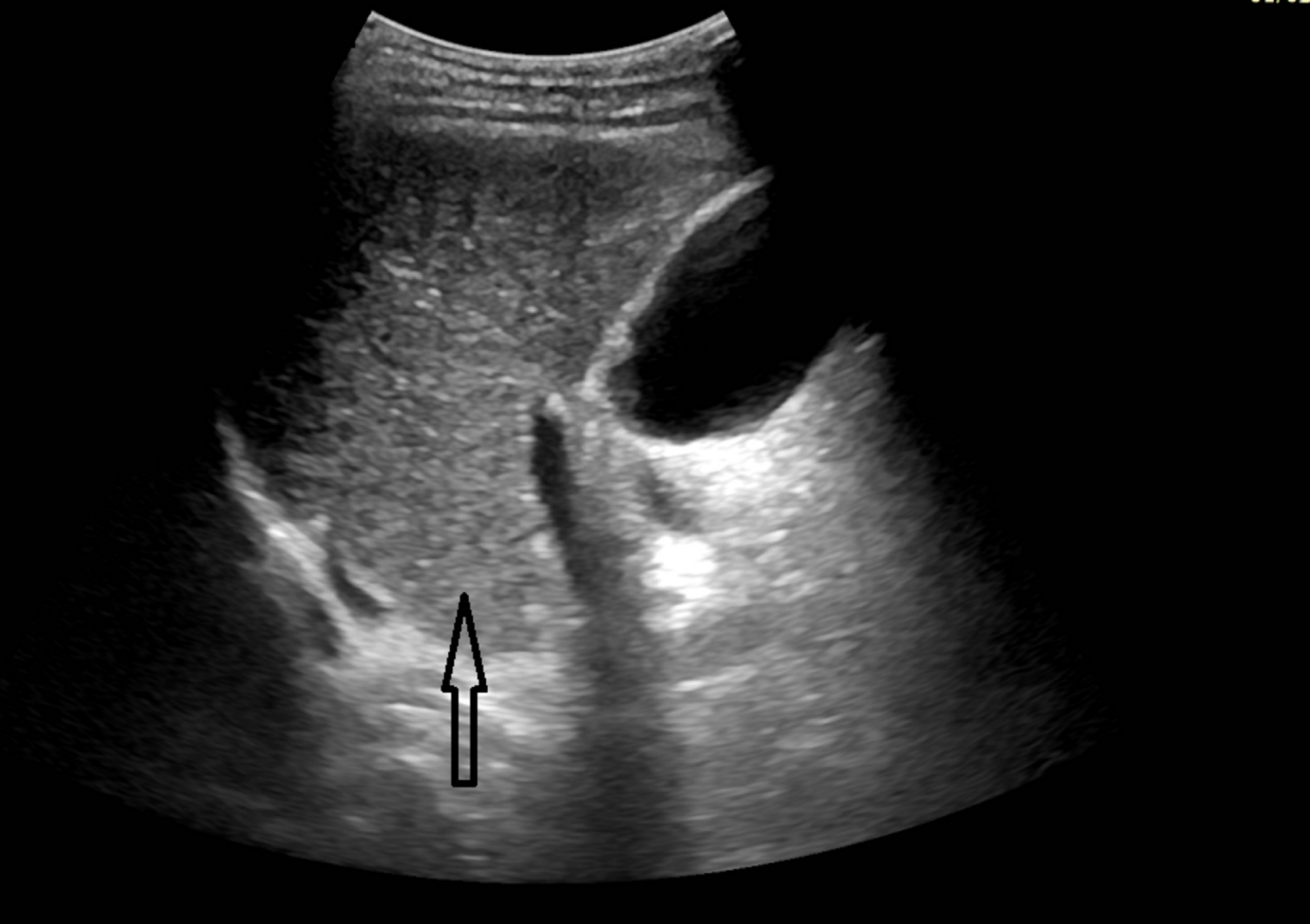
The arrow displays an ultrasound section through the right oblique parasaggital plane of liver demonstrating slightly coarse and heterogeneous liver parenchyma.

The U/S of the liver showed a slightly coarse and heterogeneous liver parenchyma along with mild splenomegaly. The slightly coarse and heterogeneous liver parenchyma is shown in Figure 1. 

**Figure 2:**
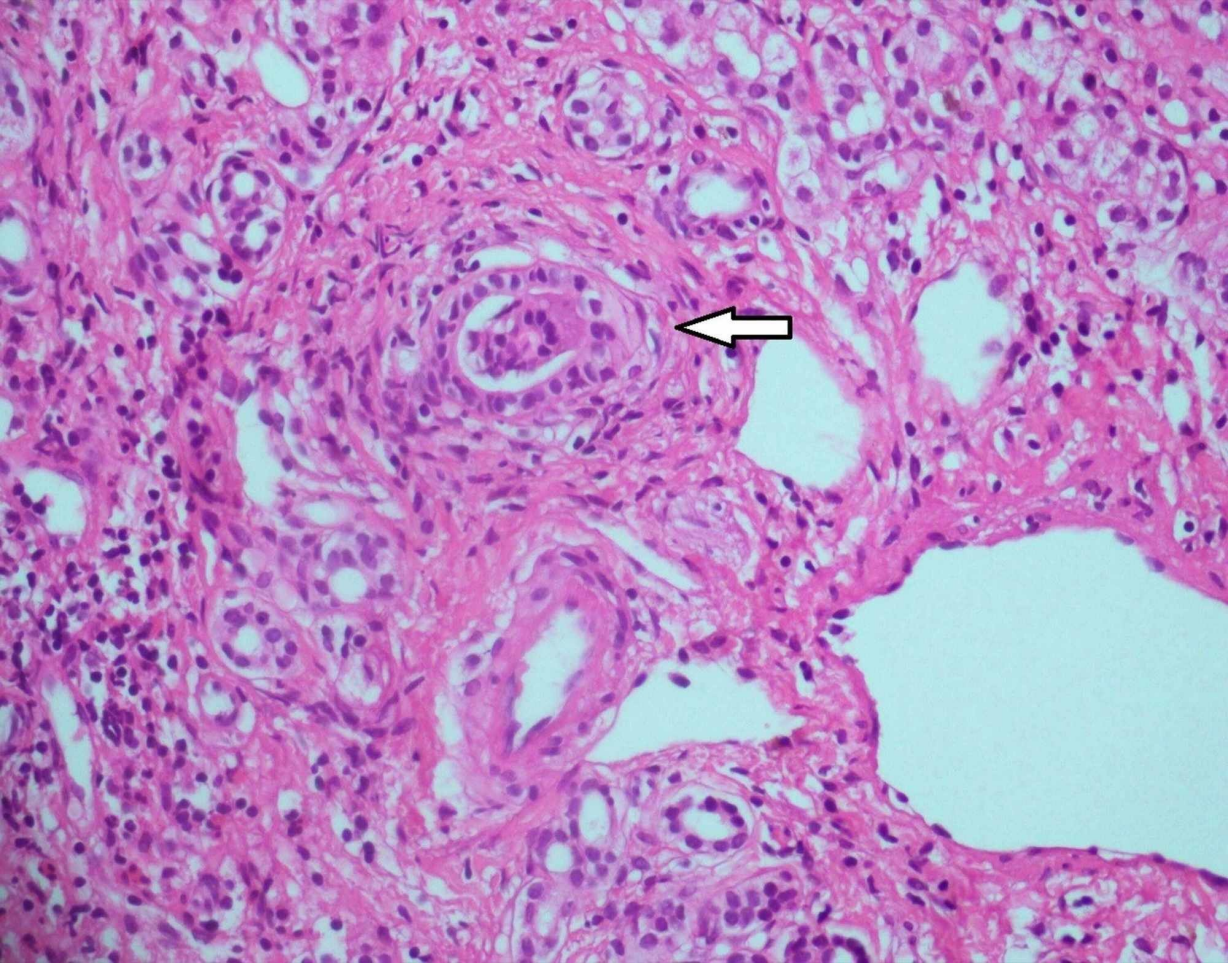
Liver biopsy shows a bile duct surrounded by onion-skinning in favor of primary sclerosing cholangitis (hematoxylin and eosin stain, original magnification ×400).

The MRCP study was normal. The U/S-guided liver biopsy showed marked ductular proliferation, with inflammatory infiltrate (lymphocytes, plasma cells and eosinophils). A few ducts revealed periductal onion-skinning suggestive of PSC (Figure 2). 

He was commenced on oral ursodeoxycholic acid with a dose of 15 mg/kg/day, fat-soluble vitamins, spironolactone and prophylactic dose of propanolol. The nutritional support was also provided. The clinical condition and laboratory investigations in terms of CLD have improved with appropriate medication given for six months. The MELD score also improved and declined from 19 to 9. He will continue to take medications in the future until he needs an liver transplant (LT). The comparison of his initial and recent laboratory investigations is mentioned in Table 1. 

**Table 1:** Initial and recent laboratory tests Hb, hemoglobin; WCC, white cell count; INR, international normalized ratio; AST, aspartate transferase; ALT, alanine transferase; ALP, alkaline phosphatase; GGT, gamma-glutamyl transferase

Laboratory Tests	Reference Range	Initial Results	Recent Results
Hb	11-17 g/dL	11	11.4
WCC	4-10 × 10^3 u/L	4.8	5
Platelets	150-450 × 10^3/u/L	90	160
INR	0.8-1.2	1.5	1.2
Total Bilirubin	0-1.2 mg/dL	5.4	1.1
Direct Bilirubin	0-0.2 mg/dL	4.4	0.7
AST	5-35 IU/L	342	47
ALT	5-45 IU/L	174	30
GGT	7-45 IU/L	75	17
ALP	54-369 IU/L	494	333
Albumin	3.5-5 g/dL	1.7	3.1

**Figure 3:**
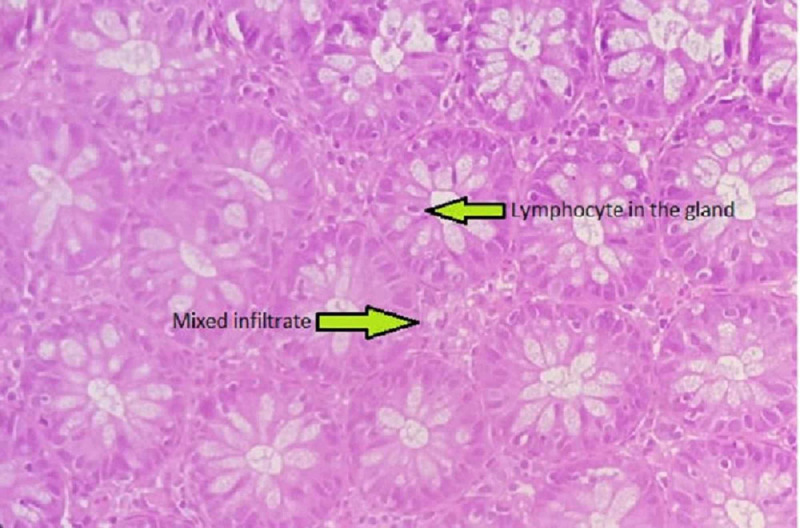
Colonic epithelium with mixed infiltrate in lamina propria and glands are infiltrated by mature lymphocyte.

Although his liver functions improved after six months of treatment, three months later he developed intermittent abdominal pain associated with watery stools containing mucus.The esophagogastroduodenoscopy was normal. No esophageal varices were seen. Colonoscopy revealed that the entire colonic mucosa was normal. However, the mucosal biopsies were diffusely infiltrated by plasma cells, abundant lymphocytes, scattered polymorphous and eosinophils confirming lymphocytic colitis (LC) (Figure 3). 

He was treated with mesalazine which led to improvement in symptoms of colitis in two months.

## Discussion

Because of the lack of awareness and expertise in the developing world like Pakistan, not many tertiary centers can cater to investigate and manage children with CLD. In our case, the referral came through to us very late. His underlying disease got worse, but our systematic approach and adequate resources in a tertiary hospital enabled us to investigate and confirm PSC and associated LC in a timely manner.

After establishing cholestatic jaundice with deranged LFTs, abnormal U/S liver and excluding other common causes of CLD, MRCP was performed to rule out extrahepatic bile duct involvement [2].

PSC involves either small intrahepatic or large ducts. Small duct involvement is not picked up on MRCP and endoscopic retrograde cholangiopancreatography (ERCP); therefore, liver biopsy remains a good tool to confirm it. The great improvement and excellent results in MRCP lately have substituted ERCP in establishing PSC. ERCP is generally required in children after some positive and suggestive findings of strictures secondary to PSC. The rate of success and complications of ERCP in children could be same as of with adults [3].

In our patient, ERCP was not performed due to unremarkable MRCP study. Because of normal MRCP, liver biopsy was performed which showed the typical features of PSC that helped us in establishing the diagnosis.

Children with inflammatory bowel disease (IBD), especially ulcerative colitis, may have PSC in 10% of cases. IBD is present in 70%-80% of patients who have PSC. PSC may occur before the onset or coexist with, or may manifest post IBD [4]. 

Our patient developed colitis a few months after establishing diagnosis of PSC. Macroscopic picture of colon was normal, but the biopsies were in favor of LC. LC (a type of microscopic colitis) is considered to be a rare association of PSC [5].

Microscopic colitis is very rare in children that links chronic watery diarrhea with intraepithelial lymphocytosis (LC) or accumulation of subepithelium collagen (collagenous colitis). Unlike IBD, It does not exhibit colonic mucosal damage during colonoscopy or show distortion of architecture of crypts and loss of goblet cells in biopsies [6].

LT is the ultimate treatment for PSC. It almost contributes 5% of all LTs in the USA [7]. After LT prognosis remains almost the same as that of other pediatric liver diseases with more than 90% of patients and graft survival at five years. We hope that our patient may not need LT for years as he has improved with help of necessary workup and further management.

## Conclusions

Children with suspected CLD should be referred to tertiary centers promptly to avoid any delay in making diagnosis to initiate appropriate treatment, especially in developing countries like Pakistan. Early pick up and referral would not only slow the progression of disease but also delay liver transplantation for years.
